# The role of efflux transporters and metabolizing enzymes in brain and peripheral organs to explain drug‐resistant epilepsy

**DOI:** 10.1002/epi4.12542

**Published:** 2021-10-01

**Authors:** Marta Vázquez, Pietro Fagiolino

**Affiliations:** ^1^ Pharmaceutical Sciences Department Faculty of Chemistry Universidad de la República Montevideo Uruguay

**Keywords:** efflux transporter overexpression, iatrogenic refractoriness, L‐carnitine deficiency, metabolizing enzyme overexpression, pharmacokinetic hypothesis, therapeutic management

## Abstract

Drug‐resistant epilepsy has been explained by different mechanisms. The most accepted one involves overexpression of multidrug transporters proteins at the blood brain barrier and brain metabolizing enzymes. This hypothesis is one of the main pharmacokinetic reasons that lead to the lack of response of some antiseizure drug substrates of these transporters and enzymes due to their limited entrance into the brain and limited stay at the sites of actions. Although uncontrolled seizures can be the cause of the overexpression, some antiseizure medications themselves can cause such overexpression leading to treatment failure and thus refractoriness. However, it has to be taken into account that the inductive effect of some drugs such as carbamazepine or phenytoin not only impacts on the brain but also on the rest of the body with different intensity, influencing the amount of drug available for the central nervous system. Such induction is not only local drug concentration but also time dependent. In the case of valproic acid, the deficient disposition of ammonia due to a malfunction of the urea cycle, which would have its origin in an intrinsic deficiency of L‐carnitine levels in the patient or by its depletion caused by the action of this antiseizure drug, could lead to drug‐resistant epilepsy. Many efforts have been made to change this situation. In order to name some, the administration of once‐daily dosing of phenytoin or the coadministration of carnitine with valproic acid would be preferable to avoid iatrogenic refractoriness. Another could be the use of an adjuvant drug that down‐regulates the expression of transporters. In this case, the use of cannabidiol with antiseizure properties itself and able to diminish the overexpression of these transporters in the brain could be a novel therapy in order to allow penetration of other antiseizure medications into the brain.

**Key points**

•Different hypotheses explaining drug‐resistant epilepsy converge toward a reduction in drug concentration at the action site.
•Some antiseizure medications lead to a very probable genesis of drug‐resistant epilepsy
•Once‐daily dosing of phenytoin and coadministration of carnitine with valproic acid could avoid iatrogenic refractoriness

## 1 INTRODUCTION

Epilepsy is a common neurological disease affecting about 50 million people worldwide.^1^ Although new antiseizure medications (ASMs) have entered the market over the last decades, about one ‐third of patients with epilepsy still continue suffering from uncontrolled seizures resulting in drug‐resistant epilepsy. The International League Against Epilepsy (ILAE) Task Force proposed that “drug‐resistant epilepsy may be defined as failure of adequate trials of two tolerated and appropriately chosen and used ASM schedules (whether as monotherapies or in combination) to achieve sustained seizure freedom.”^2^ Drug‐resistant epilepsy is associated with increased morbi‐mortality, psychiatric, serious neurological and cognitive disorders, economic and social burden, and thus a reduced quality of life.^3^ Moreover, it can lead to sudden unexpected death (SUDEP).^4^

Understanding the underlying mechanism of resistance to ASMs is crucial in order to develop either new therapeutic options or different administration regimens of the well‐known ASMs. Several hypotheses have been postulated to explain drug‐resistant epilepsy such as the transporter hypothesis, the target hypothesis, the pharmacokinetic hypothesis, the gene variant hypothesis, the epigenetic hypothesis, the intrinsic severity hypothesis, the neural network hypothesis, and the neuroinflammation hypothesis. Although none of them individually explains the neurobiological and biological basis of drug‐resistant epilepsy as the mechanism is likely to be multifactorial, a typical pattern of overexpression of efflux transporters and/or metabolizing enzymes is developed in all types of epilepsy.^5^, ^6^, ^7^, ^8^ Transporters and enzymes are the ones that allow the drug to reach the sites of action. Therefore, several of the aforementioned hypotheses give rise to a new and broader pharmacokinetic perspective to explain refractory epilepsy.

When considering the most appropriate choice of treatment, it is essential to be confident about the diagnosis and the etiology of epilepsy. A rational pharmacotherapy with ASMs combinations and non‐pharmacological treatment options such as epilepsy surgery, neurostimulation such as vagus nerve stimulation, ketogenic diet among others are possible strategies to overcome drug resistance.^7^

The discussion in this review is mainly focused on this new pharmacokinetic perspective that encompasses several of the hypotheses (transporter, pharmacokinetic, gene variant, and neuroinflammation hypotheses) which can influence ASMs’ concentrations in their action sites, on transporter upregulation and/or induction of metabolizing enzymes by ASMs themselves, and on the use of transporter inhibitors to overcome such ineffective brain drug concentration. Finally, future perspectives of pharmacological treatment strategies are presented guided by the current understanding of drug‐resistant epilepsy.

## 2 A BROADER PHARMACOKINETIC PERSPECTIVE TO EXPLAIN DRUG‐RESISTANT EPILEPSY

### 2.1 Transporter hypothesis

According to this hypothesis, drug‐resistant epilepsy is due to overexpression of efflux transporters at the blood brain barrier (BBB) and/or the epileptic foci; thus, ASMs that are subject to active transport by the efflux transporters cannot reach the action site.^7^, ^9^, ^10^ Multidrug transporters such as P‐glycoprotein (P‐gp), members of the multidrug resistance protein (MRP) family, and breast cancer‐related protein (BCRP) are usually located in the BBB in order to protect the brain from lipophilic xenobiotics.^11^ These transporters are not only located in brain capillary endothelial cells of the BBB but also in the astroglial endfeet that covers the blood vessels.^12^, ^13^ Numerous studies demonstrated overexpression of efflux transporters at the BBB and astrocytic expression, the latter presenting another barrier for reducing drug uptake in epileptic tissue.^13^, ^14^, ^15^, ^16^, ^17^, ^18^, ^19^, ^20^, ^21^, ^22^, ^23^, ^24^, ^25^ Overexpression of these transporters was confirmed in several studies involving epileptogenic brain tissue of patients with drug‐resistant epilepsy.^7^, ^15^ Interestingly, these same authors also found that overexpression of these transporters, mainly Pgp, is limited to the epileptogenic tissue but not to the normal tissue nearby. However, regarding which ASMs are transported by efflux transporters at therapeutic concentrations, evidence is controversial and incomprehensive.^26^ Some studies suggested that several ASMs may be substrates for Pgp and/or MRPs. Others could not reach a consistent conclusion. This is due to the fact that different models used by several researchers (transporter‐overexpressing cell lines, transporter inhibition in cell lines and/or in animals, and transporter gene knockout mice) yielded different results.^7^ Therefore, further studies using in vivo and in vitro models and then the confirmation of the findings in patients with epilepsy are needed. The efflux transporter overexpression in the brain of patients with epilepsy can be postulated by two mechanisms: 1—the seizures themselves can induce these transporters, and/or 2—ASMs with inductive properties can cause it. Once again, with regard to the second mechanism, evidence in the literature is still inconsistent.^26^, ^27^, ^28^, ^29^, ^30^

### 2.2 Pharmacokinetic hypothesis

Regarding this hypothesis, overexpression of efflux transporters and/or metabolizing enzymes is not restricted to the brain and it can occur in peripheral organs such as intestine, liver, and kidney decreasing the amounts of ASMs available to cross the BBB.^5^, ^6^, ^7^, ^8^But two questions arise: the first one is if this overexpression in these peripheral organs is caused by the existence of seizures or by the use of ASMs with inductive properties (ie, phenytoin, carbamazepine) and the second is if overexpression in these peripheral organs always impacts in the same way on the inductive ASMs systemic concentrations.

In view of data reported by some authors, the function of the BBB is transiently and locally disrupted during seizures^12^ and that overexpression of Pgp and MRP1 was only found in the epileptogenic tissue and not in normal brain tissue of the same patients.^31^ Thus, further studies are necessary to evidence the overexpression of these transporters in peripheral organs caused by the epilepsy itself.

On the other hand, if ASMs have inductive properties on the expression of these proteins, the overexpression will also happen in peripheral organs such as intestine, liver, and kidney as the “pharmacokinetic hypothesis” suggests. According to a study carried out by our research group,^32^ induction of the expression of Pgp at different biological barriers, including the BBB, mediated by phenytoin (PHT) in a concentration‐and‐time‐dependent manner, was found when increasing oral and intraperitoneal doses of PHT were administered, to Sprague Dawley rats. Despite the low concentrations obtained after oral doses (around 7 mg/L) due to loss of bioavailability, the inductive effect of PHT on Pgp was observed in all cases and it increased with increasing doses. Interestingly, induction intensity varies among different tissues. It was higher in intestine, followed by salivary glands (acini), liver (hepatocytes and vessels), bile duct, and brain (microvessels and vessels at brain blood barrier level). After the fourth day of multiple intraperitoneal administration, the mean plasma concentration was 22.2 mg/L, concentration much higher than the one obtained after oral administrations. The immunohistochemical analysis revealed a higher Pgp expression at the brain. Therefore, it can be concluded that Pgp overexpression at the splanchnic tissues depends on the route of administration and hence on the local PHT concentration instead of the respective plasma levels, whereas efflux transporter induction at the brain is related with the plasma concentration of the inducer agent. This last fact could explain refractoriness in epilepsy treatments caused by the drug itself.^33^ Interestingly, Pgp induction was observed after the third day of administration and it returned to a basal expression seven days after the interruption of intraperitoneal treatment, whereas a longer period of time to return to basal conditions was necessary after oral treatment interruption.

Lazarowski et al^34^, ^35^, ^36^ reported subtherapeutic plasma levels of ASMs (PHT and phenobarbital) in patients with drug‐resistant epilepsy. This coincided with increased Pgp expression levels found in endothelial cells, astrocytes, and neurons from the resected brain tissue of the patients. We agreed with the authors that some drugs such as PHT, carbamazepine (CBZ), or phenobarbital can upregulate efflux transporters not only at the BBB but also in all excretory organs. This fact undoubtedly plays a critical role in the modification of both brain and systemic pharmacokinetics of the ASMs. However, plasma drug concentrations could decrease or increase with chronic administration of the drugs as they depend on the pharmacokinetics of the drugs considered, taking into account not only the transporters but also the location of the enzymes involved in their metabolism. This issue will be discussed in the next section. Nevertheless, when overexpression is imposed by the uncontrolled disease increasing the frequency of seizures, there would be no possible doses that could achieve antiseizure drug concentrations at the brain level, not even those predicted systemic concentrations, since the induction of transporters and enzymes is under intense command of the patient, not allowing the drug to exert both its therapeutic and adverse actions. This could be a reasonable explanation for the systemic overexpression of enzymes and transporters giving rise to the pharmacokinetic hypothesis postulated by Dr Lazarowski.

In other words, the so‐called pharmacokinetic hypothesis by some authors^7^, ^8^, ^36^ is closely related with the “transporter hypothesis” as changes of the expression of transporters and/or metabolizing enzymes in the peripheral organs will affect bioavailability and disposition of drugs impacting on the drug that is available to entry to the central nervous system (CNS).

### 2.3 Neuroinflammatory hypothesis

Regarding neuroinflammation, there is evidence in the literature that inflammatory processes can provoke dysfunction in the BBB.^37^, ^38^, ^39^ Furthermore, due either to brain injury or frequent seizures, the release of inflammatory mediators and glutamate by astrocytes and neurons might increase multidrug transport proteins in the BBB, contributing to resistance to some ASMs, substrates of these trasporters.^40^ Some authors found that the expression of Pgp was downregulated by a local injection of a negative regulator of interleukins of rats exposed to status epilepticus.^41^, ^42^ Thus, neuroinflammation can reduce drug entry to the CNS and hence its concentration at the action sites.

### 2.4 Gene variant hypothesis

Genetic variants hypothesis deals with polymorphisms in genes encoding drug transporters and/or phase I and II metabolizing enzymes influencing both peripheral and brain drug concentrations. However, regarding efflux transporter polymorphisms and response to ASMs treatment, the results are inconsistent. Several researchers pointed out a lack of association,^43^, ^44^, ^45^, ^46^, ^47^, ^48^ whereas two meta‐analyses suggested that the ABCC2 G1249A polymorphism is significantly associated with a decreased risk of ASM resistance.^49^, ^50^ Some authors found that ABCC2 −24C>T, 3972C>T polymorphisms, and one ABCC2 haplotype was associated with ASMs resistance, whereas ABCC2 1249G>A and ABCB1 3435C>T polymorphisms were not associated with ASMs resistance in Chinese patients with epilepsy.^51^ Different confounding factors might avoid reaching a conclusive interpretation mainly the fact that not all the ASMs are substrates of the same efflux transporters or transported to the same extent as some authors suggested.^7^ Moreover, it should be highlighted the complexity of the possible role of polymorphisms in ASMs response in different ethnic populations. Therefore, these findings need to be confirmed with further better‐designed studies. Anyway, according to these studies some populations are prone to develop pharmacoresistance because of a higher susceptibility of expressing efflux transporters. Once more, restricted brain access of ASMs seems to be the main cause of their ineffectiveness.

On the other hand, variations in genes encoding enzymes involved in drug metabolism often affect clinical response to a very high extent. Since most drugs undergo oxidative biotransformation mediated by cytochrome P450 (CYP) enzymes, accounting for 90% of drug metabolism, polymorphisms in CYPs enzymes play a major role being an important determinant of drug concentration. Phenotypically, a specific population is composed of ultrarapid metabolizers (UMs), rapid metabolizers (RMs), normal metabolizers (NMs), intermediate metabolizers (IMs), and poor metabolizers (PMs). Thus, according to the phenotype, variability of drugs concentrations could lead to different responses that are going to be discussed in another part of this section. Among the CYP isoforms in which polymorphisms on the encoding genes exhibit a relevant effect on their activity, are the CYP2C9 and CYP2D6 isoforms.^52^ CYP2C19 presents fewer polymorphisms, and because the frequencies of variant alleles of CYP3A4 are very low, it contributes to variability mostly due to inducibility or inhibition rather than to polymorphisms. CYP3A5 is polymorphically expressed in the liver, small intestine, and kidney and represents 5 to 85% of the total hepatic and intestinal CYP3A4/5 content.^53^, ^54^

PHT is mainly metabolized by CYP2C9, so low or high activity of this enzyme is associated with decreased or increased PHT clearance, respectively, thus higher or lower plasma levels.^55^, ^56^ Similarly, genetic influences on phenobarbital metabolism are related mostly to CYP2C19 polymorphism. Therefore, patients with low activity of this enzyme showed a reduction in phenobarbital clearance.^57^

Considering Phase II enzymes: uridine diphospho‐glucuronosyltransferases (UGTs), only three isoforms are involved in drug metabolism: UGT1A, UGT2A, and UBT2B. Genes encoding these UGT enzymes are also highly polymorphic, and despite having been less studied than polymorphisms on CYPs coding genes, the influence polymorphisms in genes coding UGT have on enzyme expression and activity is extensively documented. Indeed, UGT polymorphisms have been associated with variability in drug disposition.^58^ For example, several experiments showed that genetic polymorphisms of UGTs affect the metabolism of valproic acid (VPA).^59^, ^60^

Oxidation by oxidative enzymes of CYP superfamily often leads to the formation of reactive toxic epoxide. The microsomal epoxide hydrolase (EPHX) is an enzyme that metabolizes numerous reactive epoxide intermediates to more water‐soluble derivatives to facilitate the elimination.^61^ Polymorphisms in this enzyme could therefore affect the pharmacokinetics of ASMs, and a decrease in the detoxification of this intermediate could be the cause of adverse reactions.^62^, ^63^, ^64^ In fact, in a pharmacokinetic study carried out by our research group in healthy volunteers comparing two different dosage regimes of PHT, cutaneous reactions were detected in some subjects. The study of EPHX polymorphisms on these subjects revealed that all the individuals with cutaneous rash presented EPHX mutations (decreased activity).^65^ In the same way, glutathione S‐transferase (GST) catalyzes the conjugation of glutathione with electrophiles, generally resulting in their detoxification and facilitated elimination. Polymorphisms with effects on the GST function have been observed in human populations for several isozymes.^66^

Although CYPs enzymes are largely present in liver and intestine, they are erroneously considered expressed low in the CNS (0.5‐2 of hepatic content).^67^ Their expression in the brain is cell/region specific and this localized expression may result in similar or even higher levels than in the hepatocytes or enterocytes.^68^, ^69^, ^70^, ^71^ Brain CYP enzymes impact directly not only on drug response but also on a wide range of processes (behavior, stress, cognitive processes, learning, neurotoxicity).^72^ Drug metabolizing CYPs 1A1, 2B6, 2E1, and 3A4 are found mainly in neurons,^71^ while CYP2D6 is expressed in pyramidal neurons and glial cells.^73^ CYPs are also abundant in astrocytes at BBB, helping in the regulation of drug influx into the CNS. Notably, CYP1B1 is expressed at the human BBB, and acting in conjunction with membrane transporters, they may regulate drug and xenobiotic penetration into the brain. CYP2C9 and CYP2C19 together were present mostly within the neuronal soma but with expression extending down the axons and dendrites.^74^

Regarding Phase II enzymes, a limited number of UGTs are expressed in the brain, mainly in endothelial cells and astrocytes of the BBB.^75^ An example is the presence of the UGT2B7 isoform in the brain. This isoform catalyzes the glucuronidation of morphine to 3‐O‐ and 6‐O‐glucuronide, the latter showing a higher analgesic potency than morphine but a slow transport across the BBB compared to the parent drug^75^; thus, the presence of UGT2B7 in brain may result in local formation of morphine‐6‐O‐glucuronide exerting its analgesic action.

The expression of GST enzymes is high in the human brain in comparison with UGTs.^76^

All brain enzymes of Phase I and Phase II can be induced or inhibited and can present polymorphisms and acting together with brain transporters affect pharmacokinetics of drugs in brain.

Interestingly, a remarkable feature of brain CYPs is their sensitivity to drug inducers, which may differ from induction of liver CYPs. For example, phenobarbital, a well‐known inducer, increases CYP2B expression in both the liver and brain of monkeys, whereas it increases CYP2E expression in brain, but not in liver.^77^ Human brain CYP2D6 levels are higher in smokers and alcoholics in comparison with non‐smokers and non‐alcoholics, while no change in liver CYP2D activity is detected. Codeine is metabolized by CYP2D6 to morphine. This metabolizing step is necessary for codeine‐induced analgesia. The administration of CYP2D6 inhibitors or CYP2D6 poor metabolizers produces less morphine and experiences less analgesia. An experiment carried out with rats inhibiting brain CYP2D6 but not liver with ICV propranolol decreased brain, but not plasma, morphine levels, thus decreasing analgesia after peripheral codeine administration.^78^ On the other hand, people with increased brain CYP2D activity (genetically or as a result of CYP2D induction in the CNS) may experience greater acute opioid‐induced analgesia from codeine.

An experimental study showed an overexpression of CYP3A4, CYP2C9, and CYP2E1 in human brain microvascular endothelial cells of drug‐resistant patients with epilepsy while CYP2D6 and CYP2C19 were downregulated.^79^ Another study suggested that CYP enzymes convert CBZ to a proconvulsive agent (quinolinic acid) in endothelial cells from patients with drug‐resistant epilepsy.^80^ So the pathology itself can affect CYPs activity or the anticonvulsant metabolic pattern.

**FIGURE 1:**
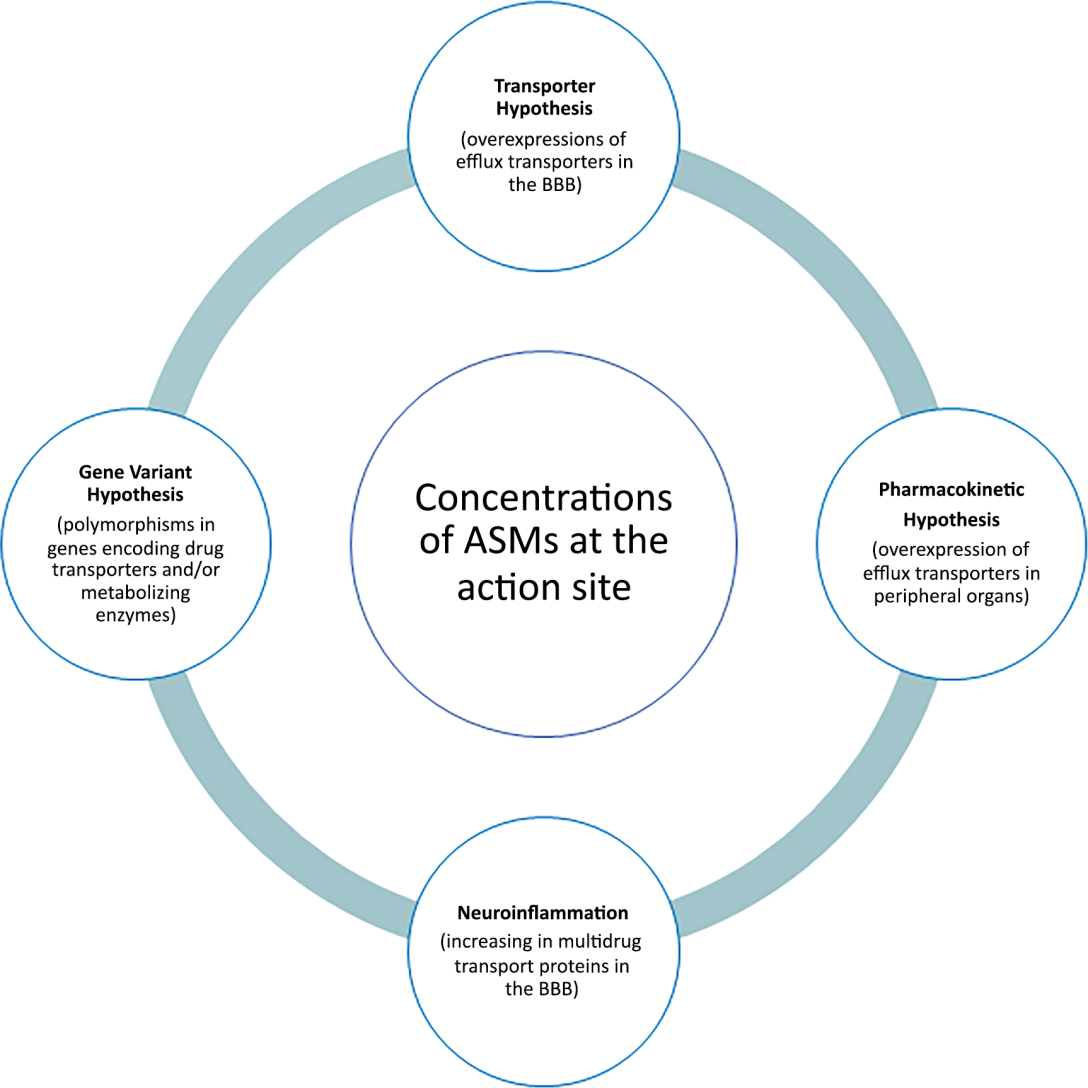
Overview of proposed hypotheses impacting on the concentration of the ASMs available on the action site

Summing up, the “transporter hypothesis,” the “pharmacokinetic hypothesis,” and even the “gene variant hypothesis” and “neuroinflammation,” among others factors, can impact on the concentration of the ASMs available at the action site to exert their effects (Figure 1). In other words, these four hypotheses converge toward a reduction in drug concentration at the site of action, thus forming a solid “pharmacokinetic” theory to explain drug‐resistant epilepsy, since pharmacokinetics is synonymous of “drug concentration.”

## 3 NONLINEAR PHARMACOKINETICS INDUCED BY ASMS THEMSELVES

**FIGURE 2:**
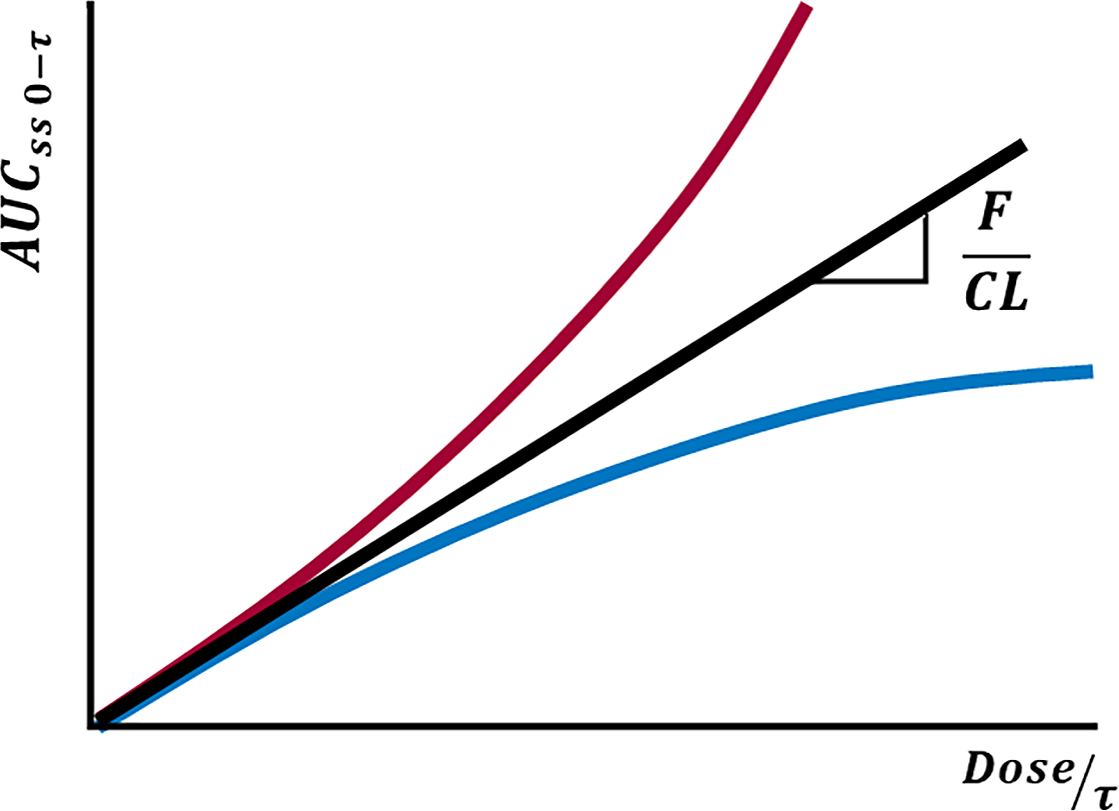
Relationships between area under the plasma drug concentration–time curve at the steady state and dose rate after multiple dose administration. Nonlinear pharmacokinetics responses are represented either by red or blue curves

Many drugs undergo nonlinear deviations on their pharmacokinetic response as dose rate increases. Nonlinearities cause changes in drug concentrations that are higher or lower to the expected from the change in dose. The most typical examples are given by CBZ (negative deviation from linearity), PHT (positive deviation), and VPA negative deviation of the total drug and positive deviation of the free drug) as shown in Figure 2.

Equation 1 correlates the area under the plasma concentration–time curve (AUC) with the dose at the steady state.

(1) $${\text{AUCss}}_{0}^{\tau }=\frac{F\cdot \text{dose}}{\text{CL}\cdot \tau }$$

Being *F* the bioavailability, τ is the drug administration interval, CL is the clearance of the drug from plasma, and thus, F/CL correlates AUC and the administration rate (dose/ τ).

PHT and CBZ show a pharmacokinetics that is time and dose dependent as they have inductive effects on both efflux transporters and metabolizing enzymes.

### 3.1 Carbamazepine

CBZ is mainly metabolized to carbamazepine‐10,11‐epoxide (active metabolite) by CYP3A4.^81^ CYP3A4 content is much higher in the small intestine enterocytes (82%) than in the liver (40%); thus, presystemic formation of the metabolite is more relevant in comparison with its systemic formation.^82^ Moreover, CBZ is a CYP3A4 inducer, and apart from being a MRP2 substrate, it also induces its expression in several peripheral eliminating organs as well as in the BBB.^81^, ^82^

When drug dosage increases, the CYP3A4 activity and efflux transporters expression increase. Consequently, the clearance of CBZ speeds up and F is reduced. In other words, F_CBZ_/CL_CBZ_ decreases with an increase in daily dose. Our research group reported a decrease in CBZ half‐life and CBZ exposure in healthy volunteers after a single dose of 400 mg of CBZ followed by multiple doses of 200 mg every 12 hours in healthy volunteers.^83^

Thus, with this drug, an increase in dose will yield a disproportionately lower concentration than expected.

### 3.2 Phenytoin

PHT is mainly metabolized by CYP2C9 and by CYP2C19 to a minor extent. These two enzymes are highly expressed in the liver but not in the enterocyte. It was proposed in the literature that the mechanism by which PHT displays its Michaelis–Menten behavior is enzyme saturation by the drug itself. But it is difficult to understand that a well‐known enzyme inducer as PHT is can provoke saturation with increasing concentrations. Thus, our group postulated another mechanism to explain this nonlinear kinetics based on the induction PHT exerts on both enzymes and efflux transporters (Pgp and MRP2).^84^ These transporters are, among other places, located in the hepatobiliar canaliculi. With increasing daily doses of PHT, efflux transporters induction is operating, extruding the drug from a site where it is extensively metabolized (liver) to a site with low content of CYP2C9/CYP2C19 (intestine), causing a progressive lowering of hepatic clearance, and thus, a disproportionated increase in plasma PHT levels as the molecules secreted to the digestive tract can be reabsorbed. Therefore, the induction of efflux transporters seems to be the cause of the nonlinearity of PHT (red curve in Figure 2).

This hypothesis of both efflux transporter and enzyme induction was observed for PHT in rats by our group^85^ with the effect caused by an efflux transport blocker as verapamil in a system in which the inductive phenomenon was already operating. The interruption of hepatobiliary transport in this state of high expression of liver enzymes could cause intense metabolization within the hepatocyte and thus lead to a significant increase in the total clearance of the drug. In other words, with the administration of verapamil, the leak that was developing from the liver during the chronic administration of PHT would be canceled as a consequence of inducing the enterohepatic circulations with subsequent reabsorption of the drug from the intestine to the systemic circulation.^85^

### 3.3 Valproic acid

VPA is highly and concentration dependent bound to plasma albumin. As VPA concentration increases, the albumin capacity to bind VPA decreases. This results in an increase in the free fraction and thus in total plasma clearance; therefore, a decrease in total plasma concentration can be observed. In other words, after a dose increase, total VPA concentration increases less than expected exhibiting a nonlinear pharmacokinetics with negative deviation (blue curve in Figure 2). However, some researchers found a positive correlation between dose rate and total plasma VPA clearance, but a negative correlation between dose rate and intrinsic (free plasma) VPA clearance in 32 patients with epilepsy under VPA monotherapy.^86^ In addition, other authors observed a decrease in the intrinsic clearance in healthy adult volunteers and a 44% increase in VPA‐free fraction when VPA dose was increased.^87^, ^88^ Therefore, and according to these observations, two dose‐dependent processes affecting VPA kinetic can be mentioned: saturable protein binding and a change in the metabolic pattern of VPA with increasing doses. These two processes lead to a nonlinear pharmacokinetics of total and free VPA: a negative deviation from the linearity when the saturable protein binding process is taking place (blue curve) and a positive deviation from linearity (red curve of Figure 2) when a change in the metabolic pattern of VPA is the cause.

VPA undergoes hepatic metabolism by three routes: β‐oxidation, as any other fatty acid, in the mitochondria, which accounts for 40% of VPA biotransformation, glucuronidation (50%), and ω‐oxidation (10%) in the cytosol. Fatty acids entrance from cytosol to mitochondria is facilitated by carnitine, amino acid derivate that is obtained from the diet (75%), but it can be also synthesized endogenously from the essential amino acids.^89^ VPA can deplete carnitine stores, especially during long‐term or high‐dose treatments, through several synergic mechanisms, resulting this depletion in decreased β‐oxidation and so a decrease in VPA intrinsic clearance. This is responsible for the nonlinear pharmacokinetics that free VPA concentration exhibits.^90^, ^91^, ^92^, ^93^ The deviation from β‐oxidation in the metabolism of VPA leads to a more than proportional increase in the 4‐en‐VPA metabolite concentration as VPA dose rate increases.^88^ In other words, 4‐en‐VPA/VPA concentration ratio increases throughout the increase in VPA dose rate. This can be explained by a linear relationship between metabolite and parent drug concentrations and by an increased bioavailability of 4‐en‐VPA metabolite.

Serious secondary effects, such as weight gain and hyperammonemia, can be seen following VPA chronic treatments. Hyperammonemia can be caused on the one hand by the inhibition of carbamoyl phosphate synthetase (CPS) by 4‐en‐VPA, the toxic metabolite that is formed during ω‐oxidation, and on the other hand by carnitine depletion since the decrease in β‐oxidation causes a decrease in acetyl‐CoA production and further decrease in N‐acetyl glutamic acid (NAGA) synthesis. NAGA is an allosteric activator of CPS, resulting its reduced synthesis in an impaired urea cycle and consequently a rise in ammonia level.^90^

The role of oxidative stress in the development of drug‐resistant epilepsy has recently been pointed out.^94^ Oxidative stress promotes inflammatory intermediates, which in turn activates the overexpression of efflux transporters, being this fact, as previously explained, one of the strongest hypotheses limiting the effectiveness of ASMs. This oxidative stress could be the result of an exacerbated neuronal hyperexcitability caused by the increase in glutamate (excitatory neurotransmitter) as a result of the high levels of ammonia that carnitine deficiency causes.^95^ However, not only glutamate but also the production of glutamine, substance that causes edema at the brain level, would be the intermediate factors in this cascade of events that is initiated by the deficient presence of carnitine in the patient.^90^

Malfunction of the urea cycle because of an intrinsic deficiency of carnitine levels in the patient could be the origin of the refractory epilepsy. Experimental models have demonstrated the effective modulation of seizures after chronic administration of L‐carnitine.^96^ Recent reports identify L‐carnitine deficiency as a cause of intractable epilepsies.^97^, ^98^ The use of ASMs inducers of transporters and/or enzymes exacerbates the refractoriness.

Weight gain can be attributed to carnitine deficiency, as this could cause an impairment on other fatty acids oxidation. In this case, the effect on drug transport is not only the cause of the nonlinear pharmacokinetics that free plasma VPA concentration exhibits, but it also affects the disposition of fatty acids and ammonia.^90^, ^91^, ^92^, ^93^

In summary, the pharmacokinetic description of these ASMs leads to a very probable genesis of drug‐resistant epilepsy. This genesis is found between a pharmacokinetic and what can be called a “pharmacodynamic” response, in the case of VPA the deficient disposition of ammonia due to L‐carnitine depletion and in the case of CBZ and PHT the induction of enzymes and transporters. Both the hyperammonemia and the induction of protein expression are pharmacodynamic responses, which in turn cause refractoriness due to a change in their pharmacokinetic responses along the change of dose.

## 4 POSSIBLE STRATEGIES TO OVERCOME DRUG RESISTANCE

Despite the fact that numerous new ASMs have been released on the market in the last decades, 30% of patients continue having seizures that are resistant to drugs.^99^ Many attempts have been proposed in the literature in order to overcome this resistance: such as the development of new drugs nonsubstrates of these transporters; the use of efflux transporters inhibitors; the use of nanocarriers in order to avoid the active transport; the use of anti‐inflammatory drugs among others.^38^, ^100^, ^101^, ^102^, ^103^, ^104^, ^105^

However, solving this problem is still a challenging task. The hypothesis of overexpression of efflux transporters is the cornerstone that best explains the refractoriness so far. Interestingly, in the study of our group mentioned before,^32^ the intraperitoneal administration of 25mg/kg/6 h of PHT provoked a notorious higher induction of transporters in the brain compared to the same intraperitoneal dose but with a different interval (100 mg/kg/24 h), whereas a higher induction of the transporters was found in the liver and intestine. Therefore, different dosing interval of PHT but with the same input rate may impact differently in efflux transporters induction. The conclusion of this study was that PHT induction was concentration and time dependent.

Bearing this observation in mind, a two‐way, two‐period crossover study was carried out by our group comparing plasma and saliva pharmacokinetic data obtained from 6 healthy subjects after two different PHT administration regimens (600 mg every 72 h or 100 mg every 12 h during 10 days).^106^According to the results obtained, a proposal of once‐daily dosing of PHT would be preferable to administrations of twice or three times a day as the inductive power of PHT will decrease by the end of the dosing interval in the first administration regimen allowing the penetration of the drug effectively into the brain. A more frequent administration may result in continuous levels of PHT and a persistent induction of the transporters avoiding PHT entry to the brain.

VPA, which has hyperammonemia as a side effect as a result of its active depletion of L‐carnitine, is in turn a substrate for MRP2. This results in a vicious cycle of seizures without resolution. By preventing ammonia from being eliminated as urea at the liver level, glutamate is synthesized at the brain level, thus generating seizures. As VPA is removed from brain tissue by the action of membrane transporters that are overexpressed by the oxidative stress of repeated seizures, it becomes ineffective as ASM. However, this chaotic panorama that appears after high doses of VPA could be counteracted if the co‐administration of L‐carnitine (or N‐acetylcarnitine) is added to the treatment.^107^ The use of VPA or another ASM that does not induce transporters and/or enzymes, associated with L‐carnitine, is a pharmacological tool that would avoid the iatrogenic refractoriness that other inducing ASMs produce.

Cannabidiol (CBD) could be a promising agent not only because of the well‐documented therapeutic effect it exerts on some types of drug‐resistant epilepsies but also because of the decrease in the efflux transporters it provokes after chronic administration that may lead to a better entry of other ASMs in the CNS.^108^, ^109^, ^110^ However, as it is used as adjunctive therapy, drug–drug interactions may occur as CBD is highly metabolized in liver and intestine by Phase I and II enzymes, enzymes plausible to be induced by PHT or CBZ or inhibited by VPA.^108^, ^109^, ^110^, ^111^, ^112^ Furthermore, its oral bioavailability is low (6%) due to its presystemic metabolism, so the systemic concentration may be not enough to regulate the efflux transporters at the BBB.^113^, ^114^ On the other hand, CBD itself can inhibit some of the enzymes involved in ASMs metabolism.^108^, ^109^, ^110^, ^111^, ^112^ So, the impact of its use on efflux transporters already overexpressed and on the concomitant ASMs needs further investigation.

## 5 CONCLUSIONS

Drug‐resistant epilepsy remains a challenge in the treatment of epilepsy. In this review, we focus on those hypotheses that impact on the concentration of the ASMs available on the action site to exert their effects, thus on their pharmacokinetics. We also discuss several pharmacological strategies to overcome drug resistance. Based on the transporter hypothesis, one strategy to counteract drug resistance is the adjunctive use of efflux transporters down regulators with anti‐seizure activity as well, such as cannabidiol. Other strategies discussed in this review include using the old ASMs but with different dosing intervals as is the case of PHT or coadministration of L‐carnitine and VPA. There are several nonpharmacological options, such as epilepsy surgery, electrical stimulation, ketogenic diet, and gene therapy, which are not discussed here.

Overcoming drug resistance is not an easy task, and based on the available data and hypotheses, more research is needed with the aim of developing better treatment strategies to overcome drug‐resistant epilepsy.

## CONFLICTS OF INTEREST

The authors have no conflict of interest to disclose. The authors confirm that they have read the Journal's position on issues involved in ethical publication and affirm that this report is consistent with those guidelines.
